# Live longer on MARS: a yeast paradigm of mitochondrial adaptive ROS signaling in aging

**DOI:** 10.15698/mic2014.05.143

**Published:** 2014-04-23

**Authors:** Gerald S. Shadel

**Affiliations:** 1 Departments of Pathology and Genetics, Yale School of Medicine; New Haven, CT 06437-8023, USA.

**Keywords:** mitochondria, reactive oxygen species, epigenetic, hormesis, aging, ataxia-telangiectasia, signaling

## Abstract

Adaptive responses to stress, including hormesis, have been implicated in longevity, but their mechanisms and outcomes are not fully understood. Here, I briefly summarize a longevity mechanism elucidated in the budding yeast chronological lifespan model by which Mitochondrial Adaptive ROS Signaling (MARS) promotes beneficial epigenetic and metabolic remodeling. The potential relevance of MARS to the human disease Ataxia-Telangiectasia and as a potential anti-aging target is discussed.

Much of what we know about many complex cellular processes (e.g., cell cycle regulation, vesicle transport, gene expression and organelle biology), we owe to the study of the *Saccharomyces cerevisiae* model system. This now also holds true for basic mechanisms of cellular aging, where the replicative and chronological lifespan of this budding yeast models aging in dividing and post-mitotic cell populations in multicellular eukaryotes, respectively. Since an overview of the methods involved and what has been learned in these aging model systems has been reviewed recently 1, I am focusing here on a developing paradigm of mitochondrial-stress signaling as a key longevity determinant based on the study of yeast chronological life span (CLS).

Mitochondria are complex organelles at the crossroads of metabolism, apoptosis (a type of programmed cell death), and signaling. Due to their bacterial ancestry, mitochondria have retained a simple, yet essential genetic blueprint that contributes critically to one of their main functions, generation of ATP via the process of oxidative phosphorylation (OXPHOS, a.k.a. respiration) 2. For example, in mammals, mitochondrial DNA (mtDNA) is a 16.5-kb circular genome that encodes 13 of the ~80 OXPHOS complex subunits, as well as two rRNAs and 22 tRNAs needed to translate these on dedicated ribosomes in the mitochondrial matrix 3. The remaining ~1,500 resident mitochondrial proteins are encoded by genes in the nucleus and imported into the organelle during or after synthesis by cytoplasmic ribosomes. In *S. cerevisiae*, mtDNA is larger (~80 kb), encodes fewer OXPHOS genes, and contains introns, but nonetheless is essential for respiratory growth 4. Because most proteins that reside in or regulate mitochondria are encoded by nuclear genes, including those needed for mtDNA replication and gene expression 3, a complex interplay exists between the nucleus and mitochondria 5,6. The so-called "anterograde" and "retrograde" signaling pathways involved in maintaining mitochondrial biogenesis, function, and homeostasis are currently not completely understood, but have emerged as important for aging and longevity.

During OXPHOS, the mitochondrial electron transport chain (ETC) produces reactive oxygen species (ROS) when electrons are transferred to oxygen at sites in the chain prior to complex IV (cytochrome oxidase) where electrons react with oxygen to form water 7. These premature, one-electron transfers generate the free radical superoxide, which can form in either the matrix or the space between the inner and outer mitochondrial membranes (Fig. 1). In addition, there are other sites of mitochondrial superoxide production 8. Mitochondrial superoxide has several fates 9. It can react with and damage molecules directly (e.g., iron-sulfur complexes found in many enzymes), it can react with nitric oxide (NO) to produce the highly reactive oxidant peroxynitrite (and reduce availability of NO for signaling), or it can be converted to hydrogen peroxide by superoxide dismutase in the matrix (SOD2) or in the inner-membrane space (SOD1). Hydrogen peroxide can also react directly with macromolecules (e.g., can oxidize cysteine residues in proteins), can be enzymatically converted to water by various enzymes (e.g., catalase and glutathione peroxidase), or can undergo the Fenton reaction to produce the highly reactive hydroxyl radical. Collectively, superoxide, hydrogen peroxide and hydroxyl radical are ROS that have been implicated in aging primarily through their damaging functions as summarized by the "mitochondrial" and "free radical" theories of aging for which there is significant support, but also contradictory evidence 10,11,12,13. However, superoxide and hydrogen peroxide are also signaling molecules 7 and, as I will summarize, part of Mitochondrial Adaptive ROS Signaling (MARS) pathways that can, perhaps surprisingly to some, increase longevity.

**Figure 1:**
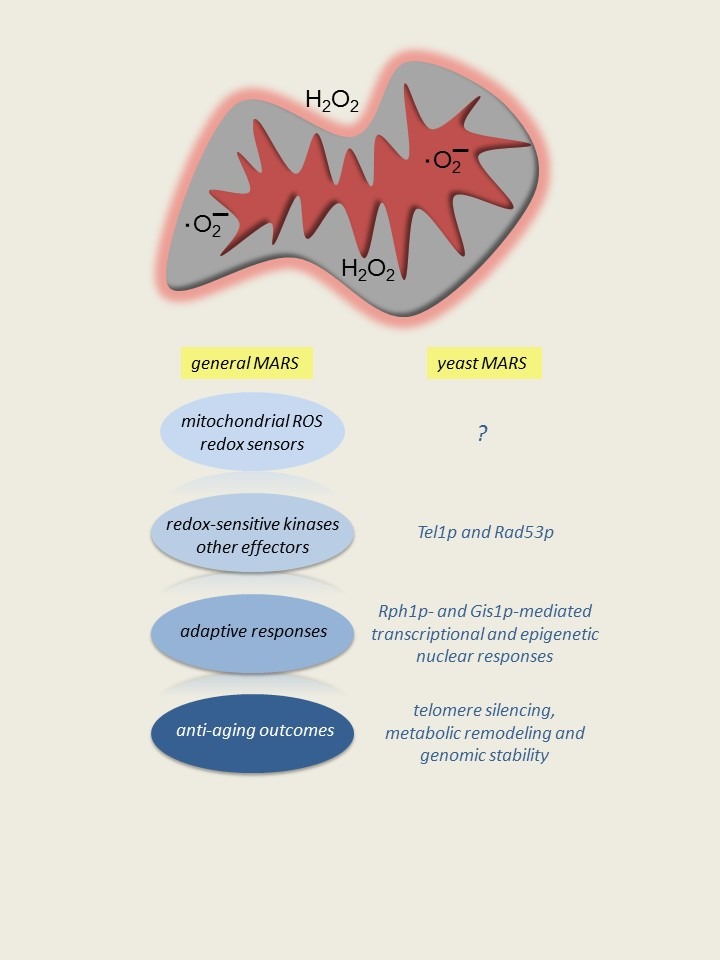
FIGURE 1: Mitochondrial Adaptive ROS Signaling (MARS). Depicted at the top are ROS (superoxide and hydrogen peroxide) generated in various mitochondrial subcompartments, and, in the case of hydrogen peroxide, possibly crossing membranes to signal directly in the cytoplasm. I propose that there are mitochondrial ROS sensors associated with mitochondria that can directly be modified by ROS or in the cytoplasm that can detect some ROS-dependent second messenger from mitochondria. MARS signals are then relayed to other cellular compartments by redox-sensitive kinases or other effectors. The end result is an adaptive change that can have a beneficial effect on cellular homeostasis and survival. To the right is the budding yeast MARS paradigm, based on CLS extension in response to reduced TORC1 signaling as described in the text. Specific known components and outcomes of yeast MARS system are shown. The question mark denotes an important gap in knowledge, which is the nature of precise mitochondrial ROS-dependent signals and sensors. A generalized MARS scheme is depicted on the left.

One conserved longevity mechanism involves reduced flux through the mechanistic target of rapamycin complex 1 (mTORC1) kinase-signaling pathway, which extends life span in many organisms 14. This pathway, which was discovered in yeast 15, stimulates pro-growth activities such as ribosome biogenesis and translation and suppresses stress responses and autophagy 16, but its anti-aging mechanism is not fully understood. We discovered that yeast TORC1 negatively regulates mitochondrial respiration in the presence of glucose and that releasing this brake on mitochondria is critical for extension of CLS 17. Interestingly, the enhanced respiration observed when TORC1 signaling is dampened is not driven by an increase in overall mitochondrial biogenesis, but rather by augmented translation of mtDNA-encoded OXPHOS subunits that results in increased density of all OXPHOS complexes in the inner membrane 18. Determining precisely how TORC1 regulates expression of mtDNA-encoded genes, controls OXPHOS density and activity, and mediates reciprocal effects on mitochondrial and cytoplasmic translation are fertile areas for future inquiry. However, we have made significant headway in understanding how the increase in mitochondrial respiration promotes extension of CLS, which brings me to explain the MARS concept below.

The dependence of CLS extension by reduced TORC1 signaling on mitochondrial respiration became more intriguing when we realized that the increase in oxygen consumption was observed only in the growth phase of culturing 17. That is, in a typical yeast CLS experiment, cells are diluted into fresh glucose medium, where they grow exponentially by fermenting glucose to ethanol. Once the glucose in exhausted, the cells switch to using this ethanol as a carbon source, which requires mitochondrial respiration (diauxic shift). After the post-diauxic phase of growth, the cells enter stationary phase, where their metabolism again changes to negotiate nutrient limitations and stress 19. Thus, the observations that CLS extension by reduced TORC1 signaling requires mitochondrial respiration and that it is increased only in the growth phase led us to postulate that a MARS response to increased respiration was driving increased survival in stationary phase (i.e. the longevity phenotype). In a nutshell, this turned out to be the case, as we recently showed that increased respiration during growth results in a mitochondrial superoxide-dependent MARS response that silences transcription at subtelomeric chromatin 20,21. This requires inactivation of the histone 3 lysine 36 (H3K36) demethylase Rph1p and hence an epigenetic response to mitochondrial ROS (mtROS) is required for the observed CLS extension 21. Interestingly, this MARS response is recapitulated using low levels of menadione (provitamin K) to produce signaling levels of mitochondrial superoxide even in the absence of increased respiration, indicating that MARS signaling per se is a key element of how reduced TORC1 signaling extends CLS 21.

The Rph1p histone demethylase is phosphorylated and inactivated by the kinase Rad53p, which is subservient to the upstream kinases Mec1p and Tel1p in the nuclear DNA-damage response 22. Accordingly, we found that the MARS pathway described above is dependent on Rad53p 21. However, mtROS signal unilaterally through Tel1p and this is not associated with a canonical DNA-damage response 21. Thus, MARS signaling in this context co-opts elements of the DNA-damage sensing machinery to relay a different type of cellular stress response, in our case, an extension of cellular life span. It remains to be determined if similar MARS signaling is involved in lifespan regulation and/or mediates some of the longevity or healthspan benefits afforded by reduced mTORC1 signaling in mammals. However, it is noteworthy that the human homolog of Tel1p is ATM, the gene for which is mutated in the inherited disease Ataxia-Telangiectasia (A-T) 23, suggesting MARS-like pathways could be involved in the etiology of this disorder. In fact, an exciting breakthrough in the biology of ATM was the realization that it not only responds to nuclear DNA damage, but also is redox sensitive (e.g., dimerizes in response to oxidization) and signals differently in response to oxidative stress versus double-strand breaks 24,25. Furthermore, we and others have described mitochondrial defects in A-T patient cells and mouse models of the disease 26,27,28,29, with reducing mtROS having some beneficial effects on the pathology observed in the latter 30. An intriguing possibility is that in the absence of the ROS-sensing function of ATM, mtROS production is not kept in check and this leads to the well-documented oxidative stress in A-T 31. This situation would likely be compounded by the inability to properly respond to and repair nuclear DNA damage, and based on recent results also mtDNA 32,33. I also speculate, based on the yeast MARS paradigm discussed, that lack of ATM might also alter epigenetic regulation and nuclear gene expression due to a role in modifying chromatin in response to mtROS in addition to mediating canonical nuclear DNA-damage responses.

As already discussed, MARS works through an epigenetic mechanism that silences subtelomeric transcription to extend yeast CLS 21. What remains to be determined is the importance of silencing subtelomeric chromatin per se for yeast longevity. That there are specific pro-aging genes located here or that the silencing is part of a response to protect telomeres and preventing nuclear genome instability (e.g., gross chromosomal rearrangements) are two possibilities that are not mutually exclusive. Increased error-prone repair and replication stress have been shown to contribute to CLS 34,35, perhaps consistent with subtelomeric silencing stabilizing the nuclear genome to extend CLS. It is also important to point out that extension of CLS by reduced TORC1 signaling is multifaceted, with subtelomeric silencing being only one key component. For example, increased stress resistance, metabolic adaptations, and other cellular processes are clearly involved 36,37,38. Many of these pathways require activation of the stress-responsive transcription factors, Msn2p/4p and Gis1p 39, the latter of which is a paralog of Rph1p that also contributes to the MARS response 21. Furthermore, Rph1p and Gis1p have been implicated in metabolic regulation (e.g., glycerol and acetate metabolism) 40,41. Interestingly, all of these factors bind to similar *cis*-acting elements 40,42 and hence likely collaborate and cross-talk to integrate various stress and metabolic inputs into transcriptional and epigenetic responses that mediate the beneficial effects of reduced TORC1 signaling.

Metabolic and epigenetic responses are burgeoning areas in aging research. In the yeast MARS paradigm (Fig. 1), the possibility that these are intimately intertwined through the Jumonji demethylase Rph1p is intriguing. Rph1p is in the dioxygenase class of histone demethylases 43 that requires iron, α-ketoglutarate and oxygen for catalysis with succinate and C0_2_ as products. In yeast, transcriptional responses that mediate mitochondrial biogenesis and function in response to oxygen have long been known to occur through heme-activated transcription factors 44,45, intimately linking nuclear transcriptional responses to mitochondrial heme biosynthesis, which requires iron. Mitochondria are also major sites of iron-sulfur center production for the ETC and many other enzymes in the cell, and their assembly and function are very sensitive to superoxide 46. Thus, iron availability may be a mechanism to signal mitochondrial function/dysfunction to the nucleus by modulating Rph1p activity. This basic concept has been proposed to contribute to nuclear genome instability downstream of mitochondrial dysfunction, based on iron-sulfur center deficiency in nuclear DNA-repair enzymes 47. Rph1p requires α-ketoglutarate and produces succinate, intermediates of the TCA cycle, potentially providing a direct link to mitochondrial metabolic activity similar to iron. Similar arguments can be posed for the sirtuins, which require the metabolic co-factor NAD^+^, a major link to the mitochondrial ETC that utilizes NADH from the TCA cycle to drive respiration, and for histone acetyltransferases that utilize the central metabolic intermediate acetyl-CoA for catalysis. This linkage of metabolism to chromatin remodeling is clearly worth significant more attention with regard to regulation of nuclear gene expression and DNA stability, in general, and to aging, specifically.

Lastly it is important to emphasize that the MARS paradigm for yeast CLS regulation I have summarized here was not elucidated in a vacuum. That is, MARS pathways in aging in *C. elegans* and yeast have been documented clearly by others 48,49,50,51. In addition, adaptive responses to other forms of mitochondrial dysfunction that regulate longevity 52, particularly the mitochondrial unfolded protein response 53, which can even act cell-non-autonomously 54, are firmly established. Therefore, I conclude that significant future efforts should be aimed at understanding mitochondrial stress-signaling pathways in biology, pathology and aging, including those like MARS with long-term adaptive effects. Delineating the full complement of these pathways, how they act in specific time and developmental windows, and transduce signals to effect specific beneficial outcomes (e.g., epigenetic regulation and metabolic remodeling) could have significant prophylactic or therapeutic value for mitochondrial and metabolic diseases, as well as age-related pathology.

## References

[1] Longo VD, Shadel GS, Kaeberlein M, Kennedy B (2012). Replicative and chronological aging in Saccharomyces
cerevisiae.. Cell metabolism.

[2] Shadel GS, Clayton DA (1997). Mitochondrial DNA maintenance in vertebrates.. Annual review of biochemistry.

[3] Bonawitz ND, Clayton DA, Shadel GS (2006). Initiation and beyond: multiple functions of the human
mitochondrial transcription machinery.. Mol Cell.

[4] Costanzo MC, Fox TD (1990). Control of mitochondrial gene expression in Saccharomyces
cerevisiae.. Annual review of genetics.

[5] Butow RA, Avadhani NG (2004). Mitochondrial signaling: the retrograde response.. Mol Cell.

[6] Poyton RO, McEwen JE (1996). Crosstalk between nuclear and mitochondrial
genomes.. Annual review of biochemistry.

[7] Sena LA, Chandel NS (2012). Physiological roles of mitochondrial reactive oxygen
species.. Mol Cell.

[8] Brand MD (2010). The sites and topology of mitochondrial superoxide
production.. Experimental gerontology.

[9] Winterbourn CC (2008). Reconciling the chemistry and biology of reactive oxygen
species.. Nature chemical biology.

[10] Alexeyev MF (2009). Is there more to aging than mitochondrial DNA and reactive oxygen
species?. The FEBS journal.

[11] Hekimi S, Lapointe J, Wen Y (2011). Taking a "good" look at free radicals in the aging
process.. Trends in cell biology.

[12] Liochev SI (2013). Reactive oxygen species and the free radical theory of
aging.. Free Radic Biol Med.

[13] Ristow M, Schmeisser S (2011). Extending life span by increasing oxidative
stress.. Free Radic Biol Med.

[14] Johnson SC, Rabinovitch PS, Kaeberlein M (2013). mTOR is a key modulator of ageing and age-related
disease.. Nature.

[15] Heitman J, Movva NR, Hall MN (1991). Targets for cell cycle arrest by the immunosuppressant rapamycin
in yeast.. Science.

[16] Sengupta S, Peterson TR, Sabatini DM (2010). Regulation of the mTOR complex 1 pathway by nutrients, growth
factors, and stress.. Mol Cell.

[17] Bonawitz ND, Chatenay-Lapointe M, Pan Y, Shadel GS (2007). Reduced TOR signaling extends chronological life span via
increased respiration and upregulation of mitochondrial gene
expression.. Cell metabolism.

[18] Pan Y, Shadel GS (2009). Extension of chronological life span by reduced TOR signaling
requires down-regulation of Sch9p and involves increased mitochondrial
OXPHOS complex density.. Aging.

[19] Werner-Washburne M, Braun EL, Crawford ME, Peck VM (1996). Stationary phase in Saccharomyces cerevisiae.. Molecular microbiology.

[20] Pan Y, Schroeder EA, Ocampo A, Barrientos A, Shadel GS (2011). Regulation of yeast chronological life span by TORC1 via adaptive
mitochondrial ROS signaling.. Cell metabolism.

[21] Schroeder EA, Raimundo N, Shadel GS (2013). Epigenetic silencing mediates mitochondria stress-induced
longevity.. Cell metabolism.

[22] Kim EM, Jang YK, Park SD (2002). Phosphorylation of Rph1, a damage-responsive repressor of PHR1 in
Saccharomyces cerevisiae, is dependent upon Rad53 kinase.. Nucleic Acids Res.

[23] Shiloh Y, Ziv Y (2013). The ATM protein kinase: regulating the cellular response to
genotoxic stress, and more.. Nat Rev Mol Cell Biol.

[24] Ditch S, Paull TT (2012). The ATM protein kinase and cellular redox signaling: beyond the
DNA damage response.. Trends Biochem Sci.

[25] Guo Z, Kozlov S, Lavin MF, Person MD, Paull TT (2010). ATM activation by oxidative stress.. Science.

[26] Ambrose M, Goldstine JV, Gatti RA (2007). Intrinsic mitochondrial dysfunction in ATM-deficient
lymphoblastoid cells.. Hum Mol Genet.

[27] Eaton JS, Lin ZP, Sartorelli AC, Bonawitz ND, Shadel GS (2007). Ataxia-telangiectasia mutated kinase regulates ribonucleotide
reductase and mitochondrial homeostasis.. J Clin Invest.

[28] Patel AY, McDonald TM, Spears LD, Ching JK, Fisher JS (2011). Ataxia telangiectasia mutated influences cytochrome c oxidase
activity.. Biochem Biophys Res Commun.

[29] Valentin-Vega YA, Kastan MB (2012). A new role for ATM: regulating mitochondrial function and
mitophagy.. Autophagy.

[30] D'Souza AD, Parish IA, Krause DS, Kaech SM, Shadel GS (2013). Reducing mitochondrial ROS improves disease-related pathology in
a mouse model of ataxia-telangiectasia.. Mol Ther.

[31] Ambrose M, Gatti RA (2013). Pathogenesis of ataxia-telangiectasia: the next generation of ATM
functions.. Blood.

[32] Schroeder EA, Shadel GS (2014). Crosstalk between mitochondrial stress signals regulates yeast
chronological lifespan.. Mechanisms of ageing and development.

[33] Sharma NK, Lebedeva M, Thomas T, Kovalenko OA, Stumpf JD, Shadel GS, Santos JH (2014). Intrinsic mitochondrial DNA repair defects in Ataxia
Telangiectasia.. DNA Repair (Amst).

[34] Madia F, Wei M, Yuan V, Hu J, Gattazzo C, Pham P, Goodman MF, Longo VD (2009). Oncogene homologue Sch9 promotes age-dependent mutations by a
superoxide and Rev1/Polzeta-dependent mechanism.. J Cell Biol.

[35] Weinberger M, Feng L, Paul A, Smith Jr DL, Hontz RD, Smith JS, Vujcic M, Singh KK, Huberman JA, Burhans WC (2007). DNA replication stress is a determinant of chronological lifespan
in budding yeast.. PLoS One.

[36] Cheng C, Fabrizio P, Ge H, Wei M, Longo VD, Li LM (2007). Significant and systematic expression differentiation in
long-lived yeast strains.. PLoS One.

[37] Powers 3rd RW, Kaeberlein M, Caldwell SD, Kennedy BK, Fields S (2006). Extension of chronological life span in yeast by decreased TOR
pathway signaling.. Genes Dev.

[38] Wei M, Fabrizio P, Madia F, Hu J, Ge H, Li LM, Longo VD (2009). Tor1/Sch9-regulated carbon source substitution is as effective as
calorie restriction in life span extension.. PLoS genetics.

[39] Wei M, Fabrizio P, Hu J, Ge H, Cheng C, Li L, Longo VD (2008). Life span extension by calorie restriction depends on Rim15 and
transcription factors downstream of Ras/PKA, Tor, and Sch9.. PLoS genetics.

[40] Orzechowski Westholm J, Tronnersjo S, Nordberg N, Olsson I, Komorowski J, Ronne H (2012). Gis1 and Rph1 regulate glycerol and acetate metabolism in glucose
depleted yeast cells.. PLoS One.

[41] Zhang N, Wu J, Oliver SG (2009). Gis1 is required for transcriptional reprogramming of carbon
metabolism and the stress response during transition into stationary phase
in yeast.. Microbiology.

[42] Liang CY, Hsu PH, Chou DF, Pan CY, Wang LC, Huang WC, Tsai MD, Lo WS (2011). The histone H3K36 demethylase Rph1/KDM4 regulates the expression
of the photoreactivation gene PHR1.. Nucleic Acids Res.

[43] Klose RJ, Gardner KE, Liang G, Erdjument-Bromage H, Tempst P, Zhang Y (2007). Demethylation of histone H3K36 and H3K9 by Rph1: a vestige of an
H3K9 methylation system in Saccharomyces cerevisiae?. Mol Cell Biol.

[44] Forsburg SL, Guarente L (1989). Communication between mitochondria and the nucleus in regulation
of cytochrome genes in the yeast Saccharomyces cerevisiae.. Annual review of cell biology.

[45] Zitomer RS, Lowry CV (1992). Regulation of gene expression by oxygen in Saccharomyces
cerevisiae.. Microbiological reviews.

[46] Vaubel RA, Isaya G (2013). Iron-sulfur cluster synthesis, iron homeostasis and oxidative
stress in Friedreich ataxia.. Molecular and cellular neurosciences.

[47] Veatch JR, McMurray MA, Nelson ZW, Gottschling DE (2009). Mitochondrial dysfunction leads to nuclear genome instability via
an iron-sulfur cluster defect.. Cell.

[48] Schulz TJ, Zarse K, Voigt A, Urban N, Birringer M, Ristow M (2007). Glucose restriction extends Caenorhabditis elegans life span by
inducing mitochondrial respiration and increasing oxidative
stress.. Cell metabolism.

[49] Van Raamsdonk JM, Hekimi S (2009). Deletion of the mitochondrial superoxide dismutase sod-2 extends
lifespan in Caenorhabditis elegans.. PLoS genetics.

[50] Yang W, Hekimi S (2010). A mitochondrial superoxide signal triggers increased longevity in
Caenorhabditis elegans.. PLoS biology.

[51] Zarse K, Schmeisser S, Groth M, Priebe S, Beuster G, Kuhlow D, Guthke R, Platzer M, Kahn CR, Ristow M (2012). Impaired insulin/IGF1 signaling extends life span by promoting
mitochondrial L-proline catabolism to induce a transient ROS
signal.. Cell metabolism.

[52] Wolff S, Dillin A (2006). The trifecta of aging in Caenorhabditis elegans.. Experimental gerontology.

[53] Baker BM, Haynes CM (2011). Mitochondrial protein quality control during biogenesis and
aging.. Trends Biochem Sci.

[54] Durieux J, Wolff S, Dillin A (2011). The cell-non-autonomous nature of electron transport
chain-mediated longevity.. Cell.

